# Identification of a selective DNA ligase for accurate recognition and ultrasensitive quantification of *N*^6^-methyladenosine in RNA at one-nucleotide resolution†
†Electronic supplementary information (ESI) available. See DOI: 10.1039/c7sc05233b


**DOI:** 10.1039/c7sc05233b

**Published:** 2018-02-15

**Authors:** Weiliang Liu, Jingli Yan, Zhenhao Zhang, Hongru Pian, Chenghui Liu, Zhengping Li

**Affiliations:** a Key Laboratory of Applied Surface and Colloid Chemistry , Ministry of Education , Key Laboratory of Analytical Chemistry for Life Science of Shaanxi Province , School of Chemistry and Chemical Engineering , Shaanxi Normal University , Xi’an 710062 , Shaanxi Province , P. R. China . Email: yanjingli@snnu.edu.cn ; Email: lzpbd@snnu.edu.cn

## Abstract

*N* ^6^-Methyladenosine (m^6^A) is the most frequent post-transcriptional modification in RNA, and it plays a critical role in biological processes. The functions of m^6^A remain largely unexplored due to a lack of highly sensitive methods to quantitatively determine the m^6^A modification fraction at a precise location. Here, we first reveal that T3 DNA ligase has significant selectivity towards the m^6^A modification. On the basis of the new finding, we establish an ultrasensitive quantitation assay for accurately determining m^6^A at one-nucleotide resolution in RNA. With the proposed assay, as low as 4 fM RNA containing m^6^A can be determined and the selectivity is up to 54.1-fold to discriminate m^6^A against unmodified adenosine (A). The sensitivity has been improved about 10^6^-fold so the proposed method can be successfully employed to accurately determine m^6^A in real biological samples, even in low abundance RNA.

## Abstract 2

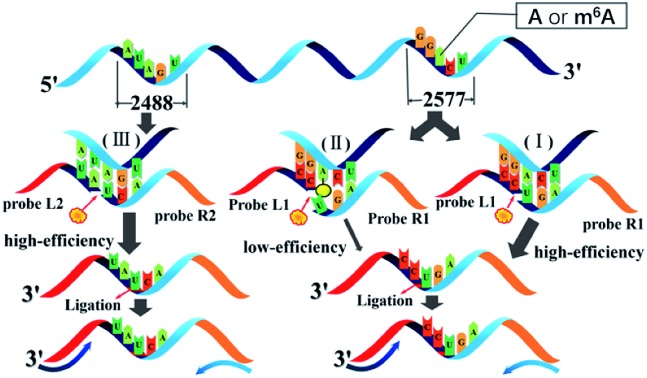
Here we establish an ultrasensitive quantitation assay for accurately determining *N*^6^-methyladenosine at one-nucleotide resolution in RNA.

## Introduction

*N* ^6^-Methyladenosine (m^6^A) is the most frequent form of post-transcriptional modification in RNA. Recent studies have shown that m^6^A occurs not only in eukaryotic and bacterial messenger RNA (mRNA)^1^,^2^ but also in viral RNA,^3^,^4^ ribosomal RNA (rRNA) and long non-coding RNA (lncRNA).^5^ The discovery of methyltransferase^6^–^9^ and demethylase^10^ for m^6^A suggests that m^6^A modification is revisable and dynamically regulated, and further indicates that m^6^A modification is associated with gene regulation, including mRNA translation efficiency,^11^ stability and splicing.^12^,^13^ Therefore, m^6^A modification plays a critical role in many biological processes and is linked to human health.^14^ However, the biology of m^6^A remains largely uncovered, due to the lack of sensitive and robust methods to quantitatively determine the degree of m^6^A modification at a precise location.

In 2012, by combining m^6^A-specific methylated RNA immunoprecipitation with massively parallel sequencing (MeRIP-seq), S. R. Jafferey’s and D. Dominissini’s groups developed high-throughput sequencing methods to analyze m^6^A modification on a transcriptome-wide scale.^15^,^16^ These studies showed that more than 7000 mammalian mRNAs contain m^6^A and that the m^6^A sites preferentially appear near stop codons and in 3′ UTRs. However, the sequencing methods cannot quantitatively detect the m^6^A modification fraction at the precise location, which generally locate the m^6^A residues within approximately 200 nucleotides. As the MeRIP-seq methods offer the distribution of m^6^A at a transcriptome-wide scale, quantitative methods for the determination of site-specific m^6^A in RNA have become increasingly important, and are greatly significant for revealing the biological functions of m^6^A and the association between m^6^A and human disease and clinical diagnosis. Liu *et al.* first reported a RNase H cleavage-based method for quantifying m^6^A at a specific site, which required site-specific cleavage and radioactive labelling followed by ligation-assisted extraction and Thin Layer Chromatography (SCARLET).^17^ The multiple enzymatic steps and the separation lead to very laborious processes, expensive reagents and radioactive hazards. More recently, E. T. Kool *et al.* have identified a DNA polymerase (Tth DNA Pol) with selectivity for the incorporation of thymidine opposite unmodified A over m^6^A.^18^ Zhou *et al.* have also revealed that Bst DNA polymerase can significantly hinder RNA-directed DNA synthesis.^19^ On the basis of the selectivity of DNA polymerases, probe extension-based methods have been established for quantifying m^6^A at specific sites. Although these methods also need electrophoresis separation and radioactive or fluorescent labels, the procedures have been simplified. Of particular note is that the sensitivity of all methods mentioned above is too low to quantify m^6^A in low abundance mRNA or lncRNA due to the lack of an amplification step, in which about 10 fmole RNA containing m^6^A can be detected in a large amount of total RNA samples. The reason may be that the products of probe extension and enzymatic cleavage are difficult to specifically amplify.

Generally, DNA ligase shows high specificity compared to DNA polymerase and the ligated DNA products can be specifically amplified. Dai *et al.* have described the T4 DNA ligase-based method to discriminate between A and m^6^A in RNA.^20^ However, no attempt was made to detect m^6^A in RNA samples, possibly due to the low selectivity of T4 DNA ligase (see below). In this work, we first reveal that T3 DNA ligase has strong selectivity to discriminate m^6^A from A in RNA. By ligating the DNA probes with T3 DNA ligase using a template of RNA and amplifying the ligated DNA products with PCR, as low as 4 fM (corresponding to 40 zmol in 10 μL solution) lncRNA containing m^6^A can be accurately determined and the selectivity for discriminating m^6^A in RNA can be greatly improved up to 54.1-fold.

## Experimental section

### Materials and reagents

RNA2577-A, RNA2577-m^6^A and RNA2488-A segments were synthesized by Integrated DNA Technologies (America). DEPC-treated water, RNAiso (lysis buffer), dNTPs and the DNA probes were obtained from TaKaRa Biotechnology Co. Ltd. (Dalian, China). JumpStart™ Taq DNA Polymerase was purchased from Sigma-Aldrich (Shanghai, China). T3 DNA ligase, T7 DNA ligase, T4 DNA ligase and Taq DNA ligase as well as T4 RNA ligase 2 were purchased from New England Biolabs (Beijing, China). Super Green I was purchased from Fanbo Biochemicals Co. Ltd. (Beijing, China). Trichloromethane, isopropyl alcohol and ethanol were purchased from Sinopharm Chemical Reagent Co. Ltd. (Beijing, China). All the solutions for reactions were prepared with DEPC-treated water.

### Ligation of DNA probes using a template of RNA

The ligation reaction mixture A consisted of 20 nM probe L, 20 nM probe R, the ligation buffer (66 mM Tris–HCl, 10 mM MgCl_2_, 1 mM ATP, 1 mM DTT, and 7.5% polyethylene glycol (PEG 6000) at pH 7.6 and 25 °C) and an appropriate amount of the RNA target. The ligation reaction mixture B consisted of 0.9 U ligase and the ligation buffer. Mixture A was heated at 85 °C for 3 min and then incubated at 35 °C for 10 min; then the ligation reaction mixture B was added. The final volume of the ligation reaction mixture was 10 μL. Then the ligation reaction mixture was incubated at an appropriate temperature to carry out ligation. After ligation, the reaction mixture was put on ice immediately. The reaction conditions for T3 DNA ligase, T4 DNA ligase, T7 DNA ligase, T4 RNA ligase 2 and Taq DNA ligase were 35 °C for 15 min, 25 °C for 30 min, 35 °C for 15 min, 37 °C for 30 min, and 35 °C for 15 min, respectively.

### PCR amplification reaction and real-time fluorescence measurements

A volume of 2 μL of the ligation product was transferred to a PCR reaction mixture with a final volume of 10 μL. The PCR reaction mixture contained a forward primer, a reverse primer (the concentration of each was 200 nM), 250 μM dNTPs, 0.4× Super Green I, 0.5 U JumpStart™ Taq DNA Polymerase and the PCR buffer (10 mM Tris–HCl at pH 8.3, 50 mM KCl, 1.5 mM MgCl_2_, and 0.001% (w/v) gelatin). The PCR reaction was carried out with a StepOne Real-Time PCR System (Applied Biosystems, USA) using a hot start of 94 °C for 2 min, followed by 45 cycles of 94 °C for 20 s and 60 °C for 30 s. The real-time fluorescence intensity was monitored at 60 °C.

### Isolation of total RNA from HeLa cells

HeLa cells were cultured in DMEM (GBICO, Cat. 12100-046) supplemented with 10% (v/v) Fetal Bovine Serum (HyClone), 100 U mL^–1^ penicillin and 100 μg mL^–1^ streptomycin. The cells were maintained at 37 °C under a humidified atmosphere containing 5% CO_2_.

The DMEM was removed from the Petri dish while the bottom of the Petri dish was filled with cells. After that the cells were washed three times with cold PBS buffer (10 mM sodium phosphate buffer, 0.1 M NaCl, at pH 7.4 and 25 °C). Next, 6 mL RNAiso solution was added into the Petri dish, and then the cells were incubated at room temperature for 2 min. After incubation, the cell lysates were collected in a centrifuge tube which contained 1.2 mL trichloromethane. The centrifuge tube was shaken slightly at room temperature for 2 min, and then the cell lysates were centrifuged for 15 min at 12000 rpm at 4 °C, and the aqueous phase was collected in a new tube. Next, 3 mL isopropyl alcohol was added to the aqueous phase and the mixture was incubated at room temperature for 10 min. Then the mixture was centrifuged for 20 min at 12000 rpm at 4 °C. The precipitate was washed once with 75% ethanol and dissolved in DEPC-treated water. Finally, the concentration of total RNA was determined with a Nano Drop 2000 (Thermo Scientific).

### Extraction of poly A^+^ RNA from total RNA

The structure of most lncRNA is similar to that of mRNA containing a polyA tail.^21^,^22^ The m^6^A to be detected is located in MALAT1 lncRNA which has a polyA tail. Therefore, MALAT1 lncRNA can be obtained by the extraction of poly A^+^ RNA.

#### Biotin-oligo (dT) binding on streptavidin (STV) of magnetic beads

First, the magnetic beads in the tube were washed three times with an STV–biotin binding buffer (150 mM KCl, 1.5 mM MgCl_2_, 10 mM Tris–HCl, 0.05 mM DDT, and 0.05% NP-40). Next, 500 μL STV–biotin binding buffer and 5 μL biotin–oligo (dT) (the concentration was 50 μM) were added into the tube. Then the tube was placed at 4 °C for 4 h with slight shaking, and thus the biotin–oligo (dT) bound onto the STV of the magnetic beads. The free biotin–oligo (dT) was washed out with washing buffer (10 mM Tris–HCl and 150 mM NaCl). Finally, 500 μL 2× binding buffer (300 mM KCl, 3 mM MgCl_2_, and 20 mM Tris–HCl) was added into the tube.

#### Hybridization with poly A^+^ RNA

The magnetic beads and total RNA (the volume was 500 μL) were heated separately at 85 °C for 3 min, and after that the magnetic beads were transferred into the total RNA and the mixture was heated at 85 °C for another 3 min. Next, the mixture was shaken for 30 min at room temperature, and during that time the 3′ poly (A) region present in poly A^+^ RNA was hybridized with biotin–oligo (dT).

#### Elution of poly A^+^ RNA

The magnetic beads were washed three times with washing buffer. Then poly A^+^ RNA attached to the magnetic beads was eluted with DEPC-treated water. The concentration of poly A^+^ RNA was determined with a Nano Drop 2000 (Thermo Scientific) and the poly A^+^ RNA was stored at –80 °C.

### Determination of melting temperatures (*T*_m_) of the hybrids of the RNA segments and the probes

To determine the melting temperature (*T*_m_) of the hybrids of RNA targets and the DNA probes in the ligation reaction, 200 nM RNA target (RNA2577-A or RNA2577-m^6^A) was mixed with 200 nM probe L1 and 200 nM probe R1 D in a 10 μL mixture solution which contained T3 DNA ligase reaction buffer (66 mM Tris–HCl, 2 mM ATP, 10 mM MgCl_2_, 1 mM DTT, and 3.75% PEG 6000) and 0.4× Super Green I. The mixture was put into a StepOne Real-Time PCR System (Applied Biosystems, USA) to determine the fluorescence signal changes when increasing the temperature from 25 °C to 95 °C. Fig. S1(a) and (b)† show the derivatives of the fluorescence intensity signal as a function of temperature, respectively, produced by RNA2577-A/(probe L1 – probe R1 D) and RNA2577-m^6^A/(probe L1 – probe R1 D). After that, probe R1 D was replaced by probe R1 and we conducted the same experiment. The derivatives of the fluorescence intensity signal as a function of temperature, produced by RNA2577-A/(probe L1 – probe R1) and RNA2577-m^6^A/(probe L1 – probe R1) are depicted in Fig. S1(c) and (d),†respectively.

## Results and discussion

### Principle of the ligation-dependent PCR method for RNA m^6^A detection

**Fig. 1:**
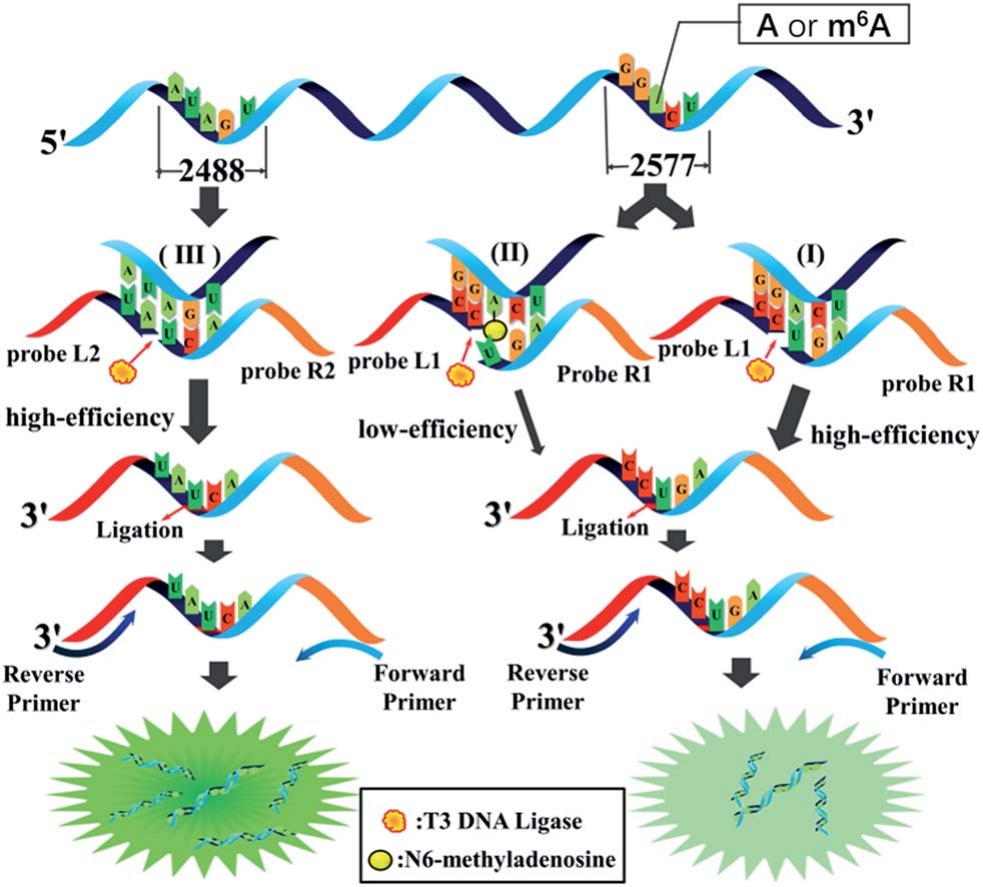
The general outline of the ligation-dependent PCR assay for m^6^A detection.

The general outline of the proposed ligase-dependent PCR assay for m^6^A determination is schematically illustrated in Fig. 1. m^6^A at the 2577^th^ site of MALAT1 lncRNA, which has been demonstrated to contain an m^6^A modification,^17^ is employed as the model target in this study. lncRNA containing m^6^A and A at the 2577^th^ site are respectively defined as RNA2577-m^6^A and RNA2577-A. For detection of RNA2577-m^6^A, DNA probe L1 (left probe) and probe R1 (right probe) were designed (see the sequence in Table S1†). Each probe contains a universal primer-specific sequence used for PCR amplification (red and orange) and a target-specific sequence (blue and black), which is respectively complementary to the RNA target immediately downstream and upstream of the 2577^th^ site. Probe L1 is modified with a phosphate group at its 5′-terminus and probe R1 is modified with two ribonucleotides at its 3′-terminus. As shown in Fig. 1(I), in the presence of RNA2577-A, probe L1 and probe R1 will adjacently hybridize with RNA around the 2577^th^ A site, and thus they can be ligated by catalysis with T3 DNA ligase. The ligated products are subsequently amplified with PCR using the universal forward primer and reverse primer. When the 2577^th^ A is methylated at the *N*^6^ position, the m^6^A at the 2577^th^ site can significantly hinder the ligation of probe L1 and probe R1 (Fig. 1(II)). Therefore, in the presence of RNA2577-m^6^A, the ligated products and final PCR products will be greatly reduced. Thus, RNA2577-m^6^A can be detected. In order to quantitatively determine the m^6^A modification fraction at the 2577^th^ site in MALAT1 lncRNA, the A at the 2488^th^ site of the same MALAT1 lncRNA molecule is selected as the reference site (Fig. 1(III)), which is known to only contain A.^15^,^16^ Probe L2 and probe R2 are designed to be the same as probe L1 and probe R1, respectively, except for the target-specific sequences, which are respectively complementary to the RNA target immediately downstream and upstream of the 2488^th^ site. In the presence of MALAT1 lncRNA, probe L2 and probe R2 will be ligated and the PCR signal of the ligated products can accurately indicate the lncRNA concentration. As demonstrated below, by comparing the real-time fluorescence PCR signals at the 2577^th^ site and 2488^th^ site, the m^6^A modification fraction can be precisely determined.

### Identification of a ligase with substantial selectivity towards m^6^A

**Fig. 2:**
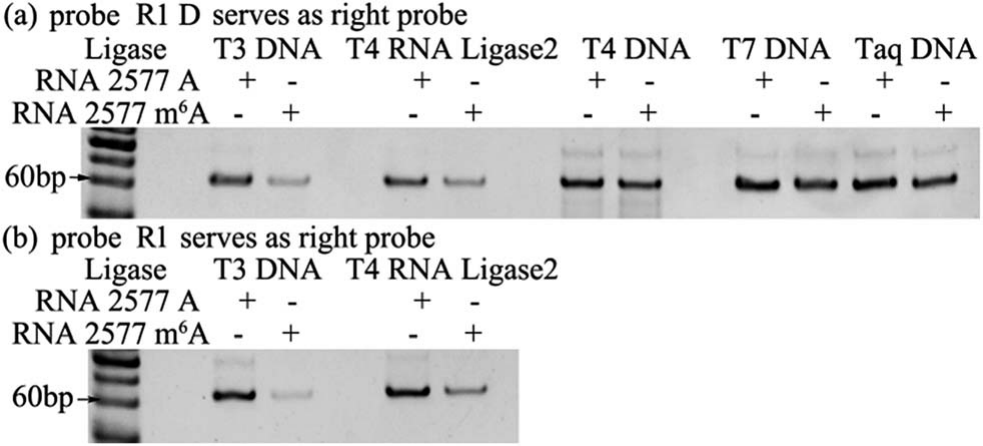
Gel electrophoresis for identifying a ligase with substantial selectivity towards m^6^A. The probes used in the reactions were probe R1 D (a) and probe R1 (b).

The selectivity of the ligases for discriminating m^6^A from A is the key issue for the ligase-dependent PCR assay to detect m^6^A in RNA. So, we first identify the substantial selectivity of the ligases towards m^6^A. A pair of 69 mer synthetic MALAT1 lncRNA segments containing either m^6^A or A at the 2577^th^ site are employed as the templates. Probe L1 and probe R1 D, which is the same as probe R1 but without modification at its 3′-terminus with two ribonucleotides, are first hybridized with the RNA2577-A or RNA2577-m^6^A segments, and are then ligated through catalysis by various ligases. The ligated products are amplified with PCR and the PCR products are detected with gel electrophoresis. As demonstrated in Fig. 2(a), when the pixel intensity of the PCR products produced from the RNA2577-A segment is defined as 100, the ratio of the pixel intensity produced from the RNA2577-m^6^A segment to that produced from the RNA2577-A segment using different ligases is 24.5 : 100 (T3 DNA ligase), 32.1 : 100 (T4 RNA ligase 2), 79.1 : 100 (T4 DNA ligase), 64.4 : 100 (T7 DNA ligase) and 77.7 : 100 (Taq DNA ligase), in which the T3 DNA ligase and T4 RNA ligase 2 show good selectivity for recognizing m^6^A from A in RNA. Using an RNA molecule as the template to ligate the DNA probes, it has been reported that modification with two ribonucleotides of one DNA probe at its 3′-terminus can greatly improve the specificity and efficiency of the ligase reaction.^23^ Therefore, probe L1 and probe R1 with the modification of two ribonucleotides at the 3′-terminus are used to further investigate the selectivity of T3 DNA ligase and T4 RNA ligase 2. As shown in Fig. 2(b), using probe L1 and R1, the ratio of the pixel intensity produced from the RNA2577-m^6^A segment to that produced from the RNA2577-A segment reaches 6.87 : 100 (T3 DNA ligase) and 30.5 : 100 (T4 RNA ligase 2). These results indicate that T3 DNA ligase has the strongest selectivity to discriminate m^6^A from A in RNA and modification of probe R1 with two ribonucleotides can greatly improve the selectivity.

The m^6^A modification is a single methylation of adenosine at the *N*^6^ position. The methyl group is small and chemically inert. More importantly, the methylated *N*^6^-position retains its ability to donate a hydrogen bond, so that m^6^A can still form Watson–Crick base pairs.^17^ Therefore, it is extremely difficult to specifically recognize m^6^A from A in RNA.^18^ Fortunately, as demonstrated above, we have revealed that different ligases have different identifying abilities towards m^6^A, and T3 DNA ligase shows the strongest selectivity, which is ascribed to its ability to recognize between m^6^A:T and A:T. We also demonstrate that T3 DNA ligase shows better selectivity to discriminate between m^6^A:U and A:U when probe R1 is modified with two ribonucleotides at its 3′-terminus. The melting temperatures (*T*_m_) of the hybrids of the RNA segments and the probes were detected, and are 44.3 °C (RNA2577-A/probe L1 – probe R1D), 43.8 °C (RNA2577-m^6^A/probe L1 – probe R1D), 43.7 °C (RNA2577-A/probe L1 – probe R1), and 41.4 °C (RNA2577-m^6^A/probe L1 – probe R1) (see Fig. S1†). Accordingly, the m^6^A modification decreases the *T*_m_ value by only 0.5 °C in the presence of m^6^A:T and A:T pairs, and in the presence of m^6^A:U and A:U pairs, the *T*_m_ value can be decreased by 2.3 °C, which also makes T3 DNA ligase more selective for recognizing m^6^A and A in RNA.

### Assessment of the effects of core sequences on specificity

**Fig. 3:**
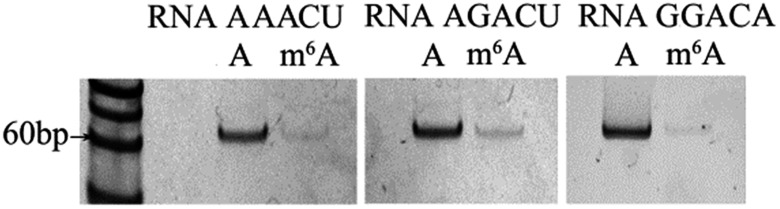
Gel electrophoresis for assessing the effects of core sequences on specificity.

In 2012, Dan Dominissini *et al.* found that all sequences with m^6^A conform to the degenerate consensus RRACH (A = m^6^A, R = A or G, H=U or A),^15^ and the commonest core sequences for m^6^A are GGACU, AAACU, AGACU and GGACA. The core sequence of RNA 2577 is GGACU. To assess the effects of the core sequences on the specificity of the proposed ligase-dependent PCR assay, the core sequence of RNA 2577 (GGACU) is replaced with AAACU, AGACU, and GGACA, respectively. The corresponding RNAs containing A or m^6^A are respectively defined as RNA AAACU-A or RNA AAACU-m^6^A, RNA AGACU-A or RNA AGACU-m^6^A, and RNA GGACA-A or RNA GGACA-m^6^A. These RNA molecules were used to perform the specificity experiments. The experiments were performed with the same procedures using T3 DNA ligase as demonstrated above. The results are shown in Fig. 3, and the ratio of the pixel intensity produced from the m^6^A segment to that produced from the A segment reaches 7.20 : 100 (RNA AAACU), 7.70 : 100 (RNA AGACU), and 5.46 : 100 (RNA GGACA). These results indicate that core sequences have little effect on the specificity of the proposed ligase-dependent PCR assay.

### Analytical performance of the ligation-dependent PCR method for RNA m^6^A detection

**Fig. 4:**
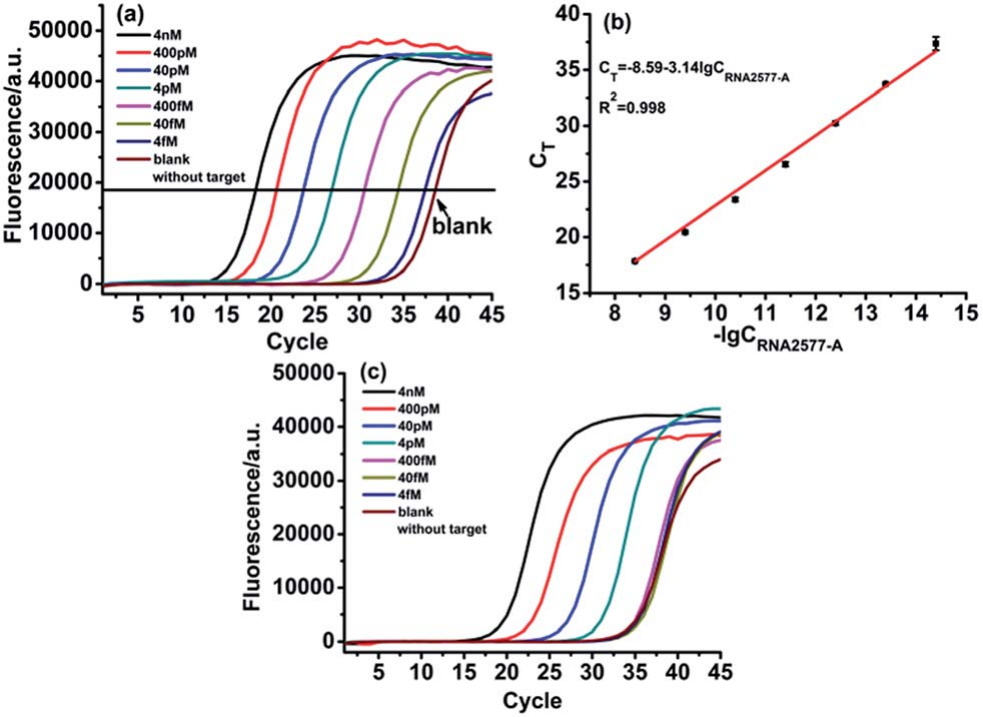
Specificity and sensitivity of the ligase-dependent PCR assay: (a) real-time fluorescence curves produced by the RNA2577-A segment in the concentration range from 4 fM to 4 nM under optimum experimental conditions. (b) The relationship between *C*_T_ and lg of RNA2577-A concentration (M). (c) Real-time fluorescence curves produced by the RNA2577-m^6^A segment in the concentration range from 4 fM to 4 nM under optimum experimental conditions.

With the proposed ligase-dependent PCR assay, we systematically investigate the influence of experimental parameters, including the ligation temperature, ligation time and amounts of T3 DNA ligase, on the selectivity and sensitivity for detecting m^6^A, and optimize the experimental conditions (see Fig. S2–S4†). Under the optimum experimental conditions, the analytical performance of the ligase-dependent PCR assay is evaluated using real-time fluorescence PCR measurements, in which Super Green I is utilized as the fluorescent dye for real-time detection of the PCR products. As shown in Fig. 4(a), well-defined real-time fluorescence curves can be obtained which are produced by the RNA2577-A segment in the concentration range from 4 fM to 4 nM. When the *C*_T_ values (the number of cycles experienced by the fluorescence signal of each reaction up to the threshold value) are plotted against the logarithm (lg) of RNA2577-A concentration, as depicted in Fig. 4(b), an excellent linear relationship is obtained. The linear regression equation is *C*_T_ = –8.59 – 3.14 lg *C*_RNA2577-A_ (M) with a corresponding correlation coefficient, *R*^2^, of 0.998. At the same time, as demonstrated in Fig. 4(c), the *C*_T_ values produced by RNA2577-m^6^A at the same concentrations are much less than those produced by RNA2577-A. According to the calibration curve shown in Fig. 4(b), the *C*_T_ values produced by 4 pM RNA2577-m^6^A correspond to the concentration of RNA2577-A at 73.9 fM. Therefore, the selectivity of the ligase-dependent PCR assay for detecting m^6^A in RNA is up to 54.1-fold. It is particularly worthwhile to note that the real-time fluorescence signal produced by 400 fM RNA2577-m^6^A is almost the same as the blank. That is to say, when the total concentration of the RNA target to be detected is less than 400 fM, RNA containing m^6^A produced a negligible signal, which will be of no contribution to the real-time fluorescence signal. The result means that the RNA-m^6^A concentration should be equal to that of the total RNA target concentration minus the RNA2577-A concentration when the total RNA concentration is less than 400 fM, which can be determined with the ligase-dependent PCR assay.

Subsequently, we tested whether the ligase-dependent PCR assay can be employed for quantitative detection of m^6^A-containing RNA. The RNA2577-A segment and RNA2577-m^6^A segment were firstly mixed with a total concentration of 400 fM as the synthetic samples, in which the RNA 2577-m^6^A proportions were 0%, 10%, 30%, 50%, 80%, and 100%. Then, the RNA2577-A concentration was determined with the ligase-dependent PCR assay according to the calibration curve shown in Fig. 4(b). The RNA2577-m^6^A concentration was equal to 400 fM minus the RNA2577-A concentration. As shown in Table S2,† the detected RNA2577-m^6^A concentrations are in good agreement with the added ones, indicating that the proposed assay can be used for quantitative evaluation of the extent of RNA containing m^6^A in RNA samples at a specific site when the total RNA concentration is less than 400 fM.

### Detection of m^6^A in real poly A^+^ RNA samples

For determination of RNA containing m^6^A in real biological samples, the vital issue is how to obtain the total concentration of the RNA target. To address this issue, as demonstrated before, we selected an adenosine (A) site in the same RNA target molecule as the reference site, which is known to only contain A, such as the 2488^th^ site in the MALAT1 lncRNA. The ligase-dependent assay can easily determine the total concentration of the RNA target molecule at the reference site, which can also control the total RNA target concentration at less than 400 fM for determination of real biological samples.

**Fig. 5:**
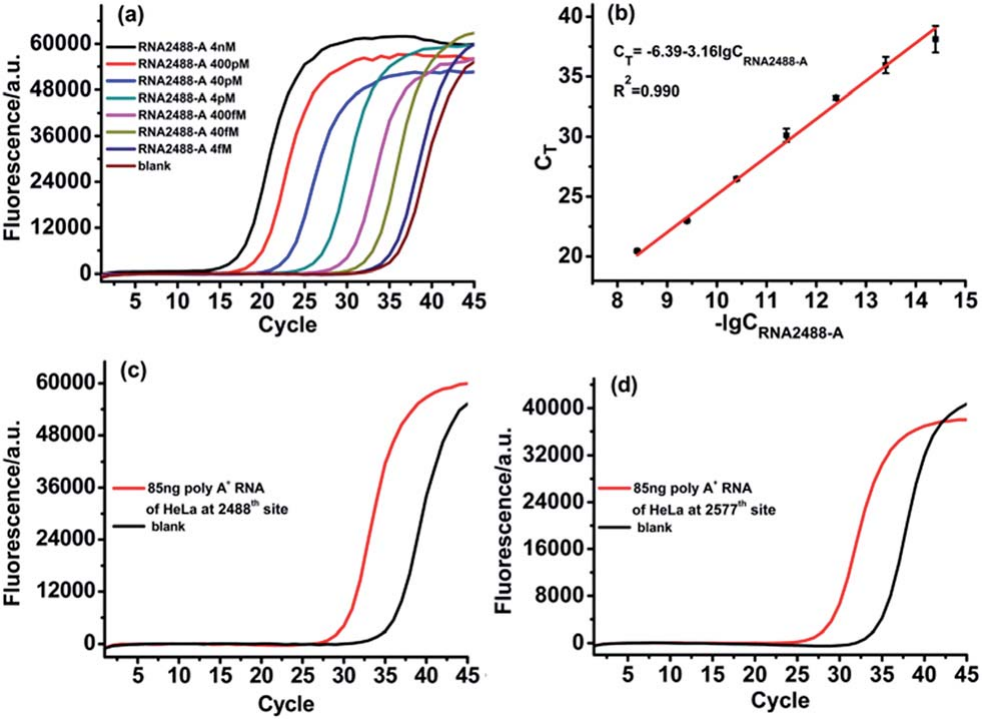
Determination of m^6^A in poly A^+^ RNA of HeLa cells. (a) Real-time fluorescence curves produced by the RNA2488-A segment in the concentration range from 4 fM to 4 nM. (b) The linear relationship between *C*_T_ and lg of RNA2488-A concentration (M). (c) Real-time fluorescence curves produced by RNA2488-A in 85 ng poly A^+^ RNA of HeLa cells under optimum experimental conditions. (d) Real-time fluorescence curves produced by RNA2577-A in 85 ng poly A^+^ RNA of HeLa cells under optimum experimental conditions.

The designed RNA2488-A specific probe L2 and probe R2 are used to perform the ligation reaction by catalysis with T3 DNA ligase and then the ligated products are amplified with PCR. A synthetic RNA2488-A segment is employed to construct the calibration curve. As demonstrated in Fig. 5(a) and (b), there is an excellent linear relationship between the *C*_T_ value and lg of RNA2488-A concentration in the range from 4 fM to 4 nM. The correlation equation is *C*_T_ = –6.39 – 3.16 lg *C*_RNA2488-A_ (M) and the correlation coefficient, *R*^2^, is 0.990. Finally, we apply the ligase-dependent PCR assay to determine the m^6^A modification fraction at the 2577^th^ site of the MALAT1 lncRNA in 85 ng poly A^+^ RNA extracted from HeLa cells. As shown in Fig. 5(b) and (c), the total concentration of MALAT1 lncRNA is determined to be 285.9 fM (*C*_RNA2488-A_) in the sample with the ligase-dependent PCR assay at the 2488^th^ site. As shown in Fig. 5(d) and 4(b), the RNA2577-A concentration is determined to be 55.2 fM (*C*_RNA2577-A_) with the ligase-dependent PCR assay at the 2577^th^ site. Therefore, the RNA2577-m^6^A concentration is calculated as 230.7 fM from the formula *C*_RNA2488-A_–*C*_RNA2577-A_, and the m^6^A modification fraction at the 2577^th^ site is estimated to be 80.7%, which very much coincides with that obtained by the SCARLET assay (∼80%).^17^ The same method is applied to the measurement of the m^6^A modification fraction at the 2577^th^ site of the MALAT1 lncRNA in poly A^+^ RNA extracted from HEK293T, and the m^6^A modification fraction at the 2577^th^ site is estimated to be 51.3% (Fig. S5†), which also coincides well with that obtained by the SCARLET assay (∼51%).^17^ The above results indicate the good applicability of the ligase-dependent PCR assay for quantitation of m^6^A in real biological samples.

## Conclusions

In summary, we have first revealed that T3 DNA ligase, a commercially available ligase, has strong selectivity for the discrimination of m^6^A from A in RNA molecules. On this basis, a highly sensitive and selective ligase-dependent PCR assay has been established, which can accurately determine the m^6^A modification fraction at any specific site in RNA. Most importantly, owing to its ultrahigh sensitivity, the proposed assay can be used to quantify m^6^A in low abundance cellular RNA in real biological samples, which is currently not possible using existing techniques due to the lack of practical ability for nucleic acid amplification. In addition, the ligase-dependent PCR assay requires only common and available lab equipment and materials. Therefore, we believe that the proposed assay should be readily promising for applications in m^6^A related fundamental studies and clinical diagnosis.

## Conflicts of interest

There are no conflicts to declare.

## Supplementary Material

Supplementary information
