# Supplementary material for: Identification of a selective DNA ligase for accurate recognition and ultrasensitive quantification of N6-methyladenosine in RNA at one-nucleotide resolution
Source: Chem Sci. 2018 Feb 15;9(13):3354–9. doi: 10.1039/c7sc05233b (PMC5932600; doi:10.1039/c7sc05233b)
Supplement: Supplementary file 1 [file SC-009-C7SC05233B-s001.pdf]

Electronic Supplementary Material (ESI) for Chemical Science.
This journal is © The Royal Society of Chemistry 2018

Electronic Supplementary Information

Identification of a Selective DNA Ligase for Accurate Recognizing and Ultrasensitive Quantifying N^6-Methyladenosine in RNAs at One-Nucleotide Resolution

Weiliang Liu, Jingli Yan,* Zhenhao Zhang, Hongru Pian, Chenghui Liu, and Zhengping Li*

Key laboratory of Applied Surface and Colloid Chemistry, Ministry of Education, Key Laboratory of Analytical Chemistry for Life Science of Shaanxi Province, School of Chemistry and Chemical Engineering, Shaanxi Normal University, Xi'an 710062, Shaanxi Province, P. R. China

Email: yanjingli@snnu.edu.cn; lzpbd@snnu.edu.cn

List of Contents:

1. Determination of melting temperature (T_m) of hybrids of RNA targets and the DNA probes

2. The effect of ligation temperature on the ligase-dependent PCR assay

3. The effect of ligation time on the ligase-dependent PCR assay

4. The effect of the amount of ligase on the ligase-dependent PCR assay

5. Determination of m^6A in poly A^+ RNA of HEK293T

Table S1. The sequences of synthetic RNA and DNA used in this study

Table S2. Quantitative determination of RNA2577-m^6A in the mixed samples

1. Determination of melting temperature (T_m) of hybrids of RNA targets and the DNA probes

Figure S1 (a) and (b) showed the derivative of the fluorescence intensity signal as the function of temperature respectively produced by RNA2577-A / (probe L1 - probe R1 D) and RNA2577-m^{6}A / (probe L1 - probe R1 D). Figure S1 (c) and (d) showed the derivative of the fluorescence intensity signal as the function of temperature respectively produced by RNA2577-A / (probe L1 - probe R1) and RNA2577-m^{6}A / (probe L1 - probe R1).

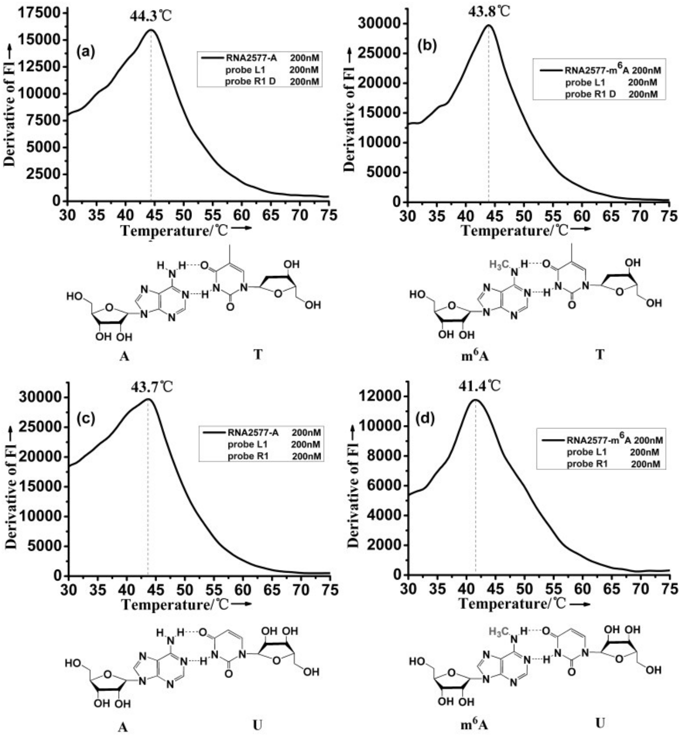

Figure S1. Determination of melting temperature (Tm) of hybrids of RNA targets and the DNA probes. (a) The Tm produced by hybrids of RNA2577-A and the DNA probes (probe L1 - probe R1 D), (b) The Tm produced by hybrids of RNA2577-m^{6}A and the DNA probes (probe L1 - probe R1 D), (c) The Tm produced by hybrids of RNA2577-A and the DNA probes (probe L1 - probe R1), (d) The Tm produced by hybrids of RNA2577-m^{6}A and the DNA probes (probe L1 - probe R1).

2. The effect of ligation temperature on the ligase-dependent PCR assay

The optimum working temperature of T3 DNA ligase is 25 °C. To investigate the influence of the ligation temperature on the ligase-dependent PCR assay, the ligation reaction were carried out at 20 °C, 25 °C, 30 °C, and 35 °C, respectively, using T3 DNA ligase. Other experiment conditions were the same as described in the Experimental Section. The blank was treated in the same way for ligation and PCR amplifications reaction just without RNA target. From Figure S2, we can see that the ligation temperature has little effect on the selectivity in the temperature range of 20°C-35 °C, but with the increasing of the temperature, the C_T values of the real-time

fluorescence curves of blank were obviously increased. Therefore, 35 °C was selected for the ligation reaction in the assay.

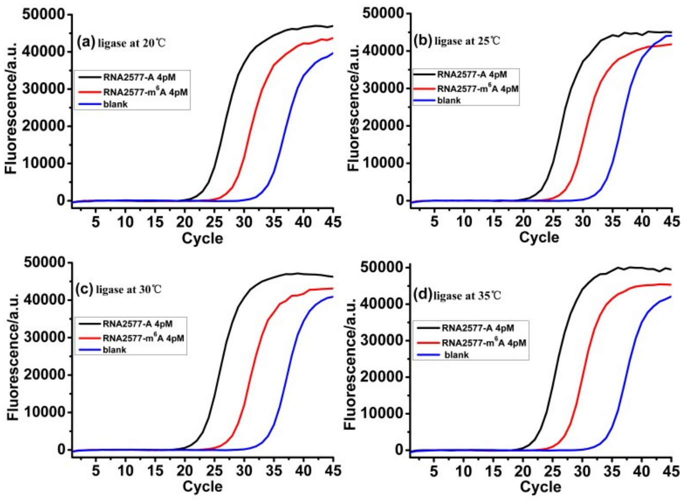

Figure S2. The effect of ligation temperature on the ligase-dependent PCR assay. The real-time fluorescence curves were produced by 4 pM RNA2577-A, 4 pM RNA2577-m^{6}A and the blank with ligase-dependent PCR assay. The ligation reactions were conducted at the following temperature (a) 20 °C, (b) 25 °C, (c) 30 °C, (d) 35 °C, respectively.

3. The effect of ligation time on the ligase-dependent PCR assay

We carried out the ligation reaction with T3 DNA ligase at 35 °C for 5 min, 15 min, and 20 min, respectively. Other experiment conditions were the same as described in the Experimental Section. The blank was treated in the same way for ligation and PCR amplification reaction just without RNA target. As depicted in Figure S3, when the ligation time were 5 min, 15 min, and 20 min, the ΔC_T between RNA 2577-A and RNA 2577-m^{6}A were 3.79, 5.20, 4.13 respectively. Therefore, the ΔC_T reaches its maximum when the ligation time was 15 min, so 15 min was chosen as the ligation time.

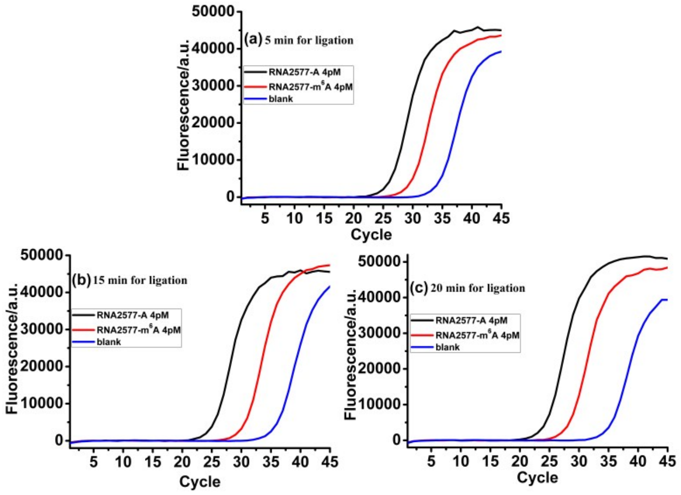

Figure S3. The effect of ligation time on the ligase-dependent PCR assay. The real-time fluorescence curves were produced by 4 pM RNA2577-A, 4 pM RNA2577-m^{6}A and the blank with ligase-dependent PCR assay. In this ligation reaction, the ligation time was (a) 5 min, (b) 15 min, (c) 20 min, respectively.

4. The effect of the amount of ligase on the ligase-dependent PCR assay

To investigate the influence of the amount of T3 DNA ligase, we conducted the ligation reaction by using 0.45 U, 0.9 U, 1.8 U and 3.6 U T3 DNA ligase, respectively. The blank was treated in the same way for ligation and PCR amplification reactions without RNA target, and other experiment conditions were the same as described in the Experimental Section. As shown in Figure S4, as the amount of T3 DNA ligase increased, the ΔC_T between RNA 2577-A and RNA 2577-m^{6}A first increased and then decreased, and the ΔC_T reaches its maximum when the ligase amount was 0.9 U, so 0.9 U T3 DNA ligase was chosen for the ligation reaction.

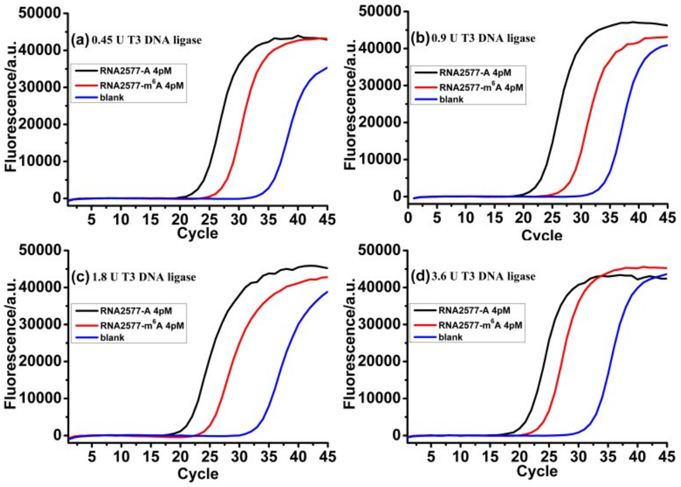

Figure S4. The effect of the amount of ligase on the ligase-dependent PCR assay. The real-time fluorescence curves were produced by 4 pM RNA2577-A, 4 pM RNA2577-m^{6}A and the blank with ligase-dependent PCR assay. The amount of T3 DNA ligase using in the ligation reaction were (a) 0.45 U, (b) 0.9 U, (c) 1.8 U, and (d) 3.6U, respectively.

5. Determination of m^{6}A in poly A$^+$ RNA of HEK293T

We applied the ligase-dependent PCR assay to determinate the m^{6}A modification fraction at 2577th site of the MALAT1 lncRNA in 70 ng polyA$^+$ RNA extracted from HEK293T cells under the optimum experimental conditions. According to the C_T value of RNA2577-A and the correlation equation C_T = -8.59 - 3.14 lg$C_{RNA2577\text{-}A}$, the RNA2577-A concentration is obtained as 88.9 fM. In the same way, according to the C_T value of RNA2488-A and the correlation equation C_T = - 6.39 - 3.16 lg$C_{RNA2488\text{-}A}$ the RNA2488-A concentration is obtained as 182.7 fM.

Therefore, the RNA 2577-m^{6}A concentration is 93.8 fM and the m^{6}A modification fraction at 2577th site is estimated to be 51.3%.

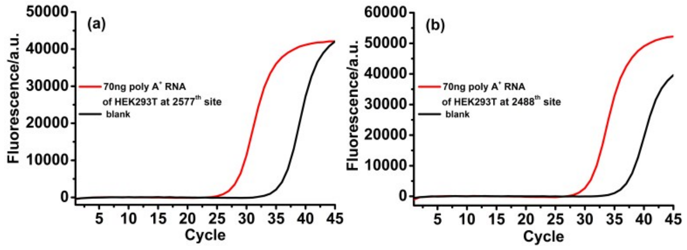

Figure S5. The determination of m^{6}A in poly A$^+$ RNA of HEK293T cells. (a).The real-time fluorescence curves produced by RNA2577-A in 70 ng poly A$^+$ RNA of HEK293T. (b) The real-time fluorescence curves produced by RNA2488-A in 70 ng poly A$^+$ RNA of HEK293T.

Table S1. The sequences of synthetic RNA and DNA used in this study.

RNA or DNA	Sequences
RNA2577-A segment	5'-CUUAAUGUUUUUGCAUUGGACUUUGAGUUAAGAUUAUUUUUUAAAUCCUGAGGACUAGCAUUAAUUGAC-3'
RNA2577-m^{6}A segment	5'-CUUAAUGUUUUUGCAUUGGm^{6}ACUUUGAGUUAAGAUUAUUUUUUAAAUCCUGAGGACUAGCAUUAAUUGAC-3'
RNA2488-A segment	5'-UAGAAGAAUUUGGAAGGCCUUAAAUAUAGUAGCUUAGUUUGAAAAAUGUGAAGG -3'
RNA AAACU-A segment	5'-CUUAAUGUUUUUGCAUUAAACUUUGAGUUAAGAUUAUU -3'
RNA AAACU-m^{6}A segment	5'-CUUAAUGUUUUUGCAUUAAm^{6}ACUUUGAGUUAAGAUUAUU -3'
RNA AGACU-A segment	5'-CUUAAUGUUUUUGCAUUAGACUUUGAGUUAAGAUUAUU -3'
RNA AGACU-m^{6}A segment	5'-CUUAAUGUUUUUGCAUUAGm^{6}ACUUUGAGUUAAGAUUAUU -3'
RNA GGACA-A segment	5'-CUUAAUGUUUUUGCAUUGGACAUUGAGUUAAGAUUAUU -3'
RNA-GGACA-m^{6}A Segment	5'-CUUAAUGUUUUUGCAUUGGm^{6}ACAUUGAGUUAAGAUUAUU -3'
Probe L1	5'-po_4CCAATGCAAAAACTCTATGGGCAGTCGGTGAT-3'[b]
Probe R1	5'-CCATCTCATCCCTGCGTGTCCTTAACTCAAArGrU-3'[a]
Probe R1 D	5'-CCATCTCATCCCTGCGTGTCCTTAACTCAAAGT-3'
Probe L2	5'-po_4ATATTTAAGGCTCTATGGGCAGTCGGTGAT-3'[b]
Probe R2	5'-CCATCTCATCCCTGCGTGTCAACTAAGCTArCrU-3'[a]
Probe L1-AAACU	5'-po_4TTAATGCAAAAACTCTATGGGCAGTCGGTGAT-3'[b]

Probe L1-AGACU	5'-po_4CTAATGCAAAAACTCTATGGGCAGTCGGTGAT-3'[b]
Probe R1-GGACA	5'-CCATCTCATCCCTGCGTGTCCTTAACTCAATrGrU-3'[a]
Forward Primer	5'-ATCACCGACTGCCCATAGAG -3'
Reverse Primer	5'-CCATCTCATCCCTGCGTGTC-3'

[a] The letter 'r' in the DNA sequences indicates ribonucleotides. [b] The 'po_4' indicates as phosphate group.

Table S2. Quantitative determination of RNA2577-m^{6}A in the mixed samples.

Sample	1	2	3	4	5	6
Added concentration of RNA2577-m^{6}A (fM)	0.0	40.0	120.0	200.0	320.0	400.0
Detected concentration of RNA2577-A (fM) [a]	402.3	365.3	281.5	202.1	81.1	12.2
Calculated concentration of RNA2577-m^{6}A (fM)	-2.3	34.7	118.5	197.9	318.2	387.8

[a] The detected results are obtained from three replicate measurements.
